# Contralateral routing of signals disrupts monaural level and spectral cues to sound localisation on the horizontal plane

**DOI:** 10.1016/j.heares.2017.06.007

**Published:** 2017-09

**Authors:** Adam J. Pedley, Pádraig T. Kitterick

**Affiliations:** a Medical Research Council, Institute of Hearing Research, The University of Nottingham, University Park, NG7 2RD, UK; b NIHR Nottingham Biomedical Research Centre, Ropewalk House, 113 The Ropewalk, Nottingham, NG1 5DU, UK; c Otology and Hearing Group, Division of Clinical Neuroscience, School of Medicine, University of Nottingham, Nottingham, NG7 2UH, UK

**Keywords:** Monaural localisation, Contralateral routing of signals (CROS), Monaural level cues, Monaural spectral cues, Unilateral deafness

## Abstract

**Objectives:**

Contra-lateral routing of signals (CROS) devices re-route sound between the deaf and hearing ears of unilaterally-deaf individuals. This rerouting would be expected to disrupt access to monaural level cues that can support monaural localisation in the horizontal plane. However, such a detrimental effect has not been confirmed by clinical studies of CROS use. The present study aimed to exercise strict experimental control over the availability of monaural cues to localisation in the horizontal plane and the fitting of the CROS device to assess whether signal routing can impair the ability to locate sources of sound and, if so, whether CROS selectively disrupts monaural level or spectral cues to horizontal location, or both.

**Design:**

Unilateral deafness and CROS device use were simulated in twelve normal hearing participants. Monaural recordings of broadband white noise presented from three spatial locations (−60°, 0°, and +60°) were made in the ear canal of a model listener using a probe microphone with and without a CROS device. The recordings were presented to participants via an insert earphone placed in their right ear. The recordings were processed to disrupt either monaural level or spectral cues to horizontal sound location by roving presentation level or the energy across adjacent frequency bands, respectively. Localisation ability was assessed using a three-alternative forced-choice spatial discrimination task.

**Results:**

Participants localised above chance levels in all conditions. Spatial discrimination accuracy was poorer when participants only had access to monaural spectral cues compared to when monaural level cues were available. CROS use impaired localisation significantly regardless of whether level or spectral cues were available. For both cues, signal re-routing had a detrimental effect on the ability to localise sounds originating from the side of the deaf ear (−60°). CROS use also impaired the ability to use level cues to localise sounds originating from straight ahead (0°).

**Conclusions:**

The re-routing of sounds can restrict access to the monaural cues that provide a basis for determining sound location in the horizontal plane. Perhaps encouragingly, the results suggest that both monaural level and spectral cues may not be disrupted entirely by signal re-routing and that it may still be possible to reliably identify sounds originating on the hearing side.

## Highlights

• Rerouting sounds from a deaf ear to a hearing ear impairs monaural localisation.
• Distinct effects of rerouting were observed on monaural level and spectral cues.
• Rerouting disrupts level cues distinguishing sounds at 0° azimuth and at the deaf ear.
• Rerouting selectively disrupts spectral cues that identify sounds at the deaf ear.

## 1 Introduction

Individuals who have access to hearing in one ear only, such as those with single-sided deafness (SSD), do not have access to the binaural cues that facilitate accurate localisation in the horizontal plane (Moore, 2012) and therefore display severely-impaired spatial hearing abilities (Colburn, 1982, Slattery and Middlebrooks, 1994). The acoustic diffraction of sound by the head (‘head-shadow effect’) can provide a basis for relatively crude judgements about the location of a sound based on its level when listening monaurally. Studies have also suggested that some monaural listeners adapt to use the effects of the outer ears (pinnae) on incoming sounds that are primarily a cue to vertical elevation (Wightman and Kistler, 1997) to distinguish sounds from different locations in the horizontal plane (Shub et al., 2008, Kumpik et al., 2010, Rothpletz et al., 2012). However, even with the use of these cues their localisation abilities remain severely-impaired relative to binaural listeners (Humes et al., 1980, Wazen et al., 2005). Substantial inter-individual variability in monaural localisation ability has been observed (van Wanrooij and van Opstal, 2004) that may relate to the presence of high-frequency hearing loss in the remaining ear (Agterberg et al., 2014).

A common audiological intervention for those with SSD is a Contralateral Routing Of Signals (CROS) hearing aid (Harford and Barry, 1965, Kitterick et al., 2014). A CROS aid comprises two hearing aid-like devices. One aid is worn on the non-hearing ear and acts as a satellite microphone for the second aid worn on the hearing ear. The acoustic coupling of this second aid is selected to be as transparent as possible to minimise occlusion of the hearing ear. The aim of this re-routing of acoustic information is to provide the listener with greater access to sound by overcoming the head shadow effect, and in doing so to aid the ability to understand speech in background noise (Hol et al., 2005). However, because the process of fitting a CROS aid attempts to minimise any differences in the acoustic signature of sounds located towards the non-hearing and hearing ears (Pumford, 2005), it possible that a well-fit CROS aid could severely restrict the availability of monaural level and spectral cues. However, empirical research does not support this conclusion.

Systematic reviews have identified six studies that have evaluated the impact of CROS use on localisation in the horizontal plane (Peters et al., 2015, Kitterick et al., 2016). Five of the six studies found no difference in localisation performance between monaural and CROS listening configurations (Arndt et al., 2011, Bosman et al., 2003, Hol et al., 2005, Hol et al., 2010, Niparko et al., 2003). However, their small sample sizes limited their statistical power to detect changes in localisation (Kitterick et al., 2016). Only one study found that the localisation abilities of CROS device users were significantly worse than those of monaural listeners (Lin et al., 2006). No study has differentiated between the effects of CROS on level or spectral cues. The conflicting nature of this evidence and the use of inconsistent methods for assessing localisation means that it is not possible to conclude whether CROS use impairs localisation ability or not (Kitterick et al., 2016).

As individuals with SSD rate spatial hearing as one of the most important listening skills that they would like to improve (McLeod et al., 2008), the current study aimed to resolve the question of whether CROS use affects localisation in the horizontal plane and if so, whether it disrupts the use of monaural level and spectral cues, or both. Although previous studies have demonstrated that acute effects of monaural listening on localisation can be induced by occluding one ear of normal hearing participants (McPartland et al., 1997, Kumpik et al., 2010, Van Wanrooij and van Opstal, 2004, Irving and Moore, 2011), the current study used monaural recordings to simulate unilateral deafness to exercise precise experimental control over the CROS fitting methodology and to minimise individual variability in high-frequency hearing thresholds that could influence access to spectral cues (Agterberg et al., 2014). It was hypothesised that: 1) with training, participants would be able to discriminate sounds from three spatially-separated locations using both monaural level cues and spectral cues; 2) by eliminating any variability in CROS fitting across participants and by ensuring the sample size was sufficiently large to achieve adequate statistical power it would be possible to demonstrate that CROS use disrupts the availability of these monaural cues and can degrade localisation performance; 3) CROS-related effects would only occur when the device was switched on as they arise due to the re-routing of signals rather than any occlusion of the hearing ear.

## 2 Methods and materials

### 2.1 Sample size

The required sample size was determined based on an *a priori* power analysis conducted using the G*Power software (Faul et al., 2007). Pilot testing with four participants suggested that the size of the effect of CROS use on monaural localisation accuracy was 1.25 standard deviations based on the specific spatial discrimination task used in the present study. To detect an effect of this size with 95% power and at α = 0.05 using a paired-sample *t*-test would require 9 participants. To account for attrition across three testing sessions, 12 participants were recruited to allow for a 25% drop-out rate whilst still achieving the desired statistical power.

### 2.2 Participants

Twelve normal-hearing adults (mean age 21.6 years, range 19–24 years) were recruited to participate. All participants reported no history of hearing problems and had pure-tone average thresholds ≤20 dB Hearing Level (HL) bilaterally, averaged across octave frequencies from 125 to 8000 Hz inclusive (mean threshold 7.2 dB HL, range 1.4–11.8). Participants received financial compensation for their participation. The study received ethical approval from the School of Psychology, University of Nottingham and all participants gave informed consent prior to data collection.

### 2.3 Stimuli recordings

Monaural recordings were made of broadband noises presented from loudspeakers located at −60°, 0°, and +60° azimuth in an anechoic chamber, where negative angles denote locations to the left of straight ahead. The noises were generated using the Matlab software package (Mathworks, Natick MA) by generating 20-sec long samples of Gaussian-distributed random noise, calculating their fast Fourier transform (FFT), setting the amplitude of components lower than 200 Hz and above 12 kHz to zero, and finally calculating the inverse FFT. This specific range of frequencies was chosen as it represented the bandwidth over which it was possible to exercise control over the output of the loudspeakers in order to achieve a flat frequency response at the listening position (Seeber et al., 2010). A pre-emphasis filter was generated for each loudspeaker by recording Maximum-Length Sequences (MLS) (Rife and Vanderkooy, 1989) at the listening position; i.e. at the point equidistant from the three loudspeakers. The filters not only ensured a flat frequency response but also equalised the output levels of the loudspeakers and synchronised the arrival times of the first wavefronts at the listening position. The pre-emphasised noises were presented using an external audio interface (MOTU 24I/O) and power amplifiers (RA150, Alesis).

Recordings of the noise stimuli were made in the right ear canal of a model listener using a probe tube microphone (Etymotic Research Inc. ER-7C Series B Clinical Probe Tube Microphone System) while they sat at the listening position. Therefore, the three spatial locations (−60°, 0°, and +60°) corresponded to the deaf side, the centre, and the hearing side, respectively. The microphone body was secured using a headband and the probe tube was inserted so that its tip was between 15 and 20 mm from the entrance of the right ear canal. The signals were high-pass filtered (3-pole Butterworth filter with −1─dB cut-off at 20 Hz) and amplified (+40 dB gain) using a battery-powered pre-amplifier (G.R.A.S. 12AK 1-Channel Power Module). The conditioned signals were sampled at 44.1 kHz with 16-bit quantization using the same external audio interface.

Three sets of monaural recordings were made. ‘Unaided’ recordings were made with the right-side ear canal open and unoccluded. The two additional sets of recordings were made while a Contra-lateral Routing of Signals (CROS) hearing aid system was worn by the model listener. The system comprised a CROS H2O satellite microphone and Cassia M H2O hearing aid (Phonak, Stefa, Switzerland), worn on the left and right ears respectively. The hearing aid was coupled to the ear canal using a slim tube and open dome to minimise occlusion, and had been fit according to manufacturer recommendations (see ‘CROS fitting’ section below). The probe tube of the microphone was inserted through one of the slits in the open dome while ensuring that the tip of the tube was positioned at least 4 mm beyond the dome to avoid near-field effects. CROS-off and CROS-on recordings were made with the devices in place with the system switched off and on, respectively.

### 2.4 CROS fitting

The CROS hearing aid system used to make the CROS-off and CROS-on recordings was fitted to the model listener who wore it following the protocol described by the manufacturer (Phonak, 2015). The fitting was achieved by connecting the CROS device to Phonak Target software (version 3.2) through a Hi-Pro programming interface (Otometrics, Taastrup, Denmark). The fitting was verified using Real Ear Measurements (REMs) via the Unity 2 fitting system (Siemens, West Sussex, UK). As the left ear was treated as the impaired ear, the satellite microphone (Phonak CROS H2O) was worn on that ear and the hearing aid (Phonak Cassia M H2O) was worn on the right ear.

**Fig. 1:**
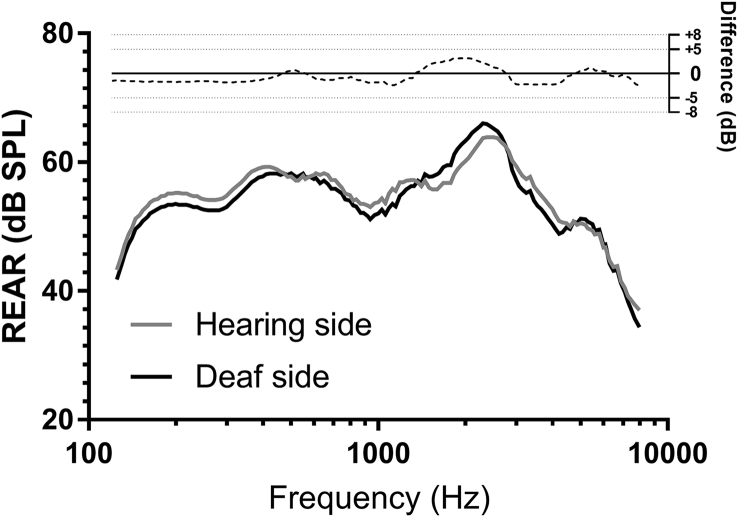
Real Ear Aided Response (REAR) measurements expressed in db Sound Pressure Level (SPL) for stimuli presented towards the deaf side (black line) and towards the hearing side (grey line) with the CROS device worn and turned on. The inset shows the difference in dB between the two measurements at each frequency and indicates that the difference was within the 5 dB (250–2000 Hz) and 8 dB (3000–4000 Hz) tolerances recommended by the British Society of Audiology (2014).

The process of fitting the CROS aid comprised three steps (Pumford, 2005): 1) A measurement was made of the response in the unimpaired ear (the right ear in this case) to a sound positioned 45° towards that ear without the CROS in place (Real Ear Unaided Response, REUR); 2) This response was re-measured but with the CROS system in place and turned on to confirm that the coupling did not effect a material change in the unaided response through occlusion (Real Ear Aided Response, REAR); 3) The REAR was measured in the right ear again but in response to a sound positioned 45° towards the other ear; i.e. towards the satellite microphone. The gain applied by the CROS aid was then adjusted to bring the response measured in step 3 to within 5 dB of the response measured in step 2 at 250, 500, 1000 and 2000 Hz and to within 8 dB at 3000 and 4000 Hz, as recommended by the British Society of Audiology (2014). The observed differences between these two aided responses at each of the measured frequencies at the end of the fitting process is shown graphically in Fig. 1.

### 2.5 Procedure

The Unaided, CROS-off, and CROS-on monaural recordings were presented to participants using an insert earphone (Etymotic ER 2) while they sat in an anechoic chamber. The output of the earphone was calibrated so that the recordings of noises from the centre location (0° azimuth) were presented at 55 dB SPL (A-weighted). This calibration was achieved by coupling the earphone to an artificial ear (Brüel & Kjær Type 4157) that was connected to a sound level meter (Apollo SINUS). The calibration apparatus was itself calibrated using a 1-kHz 94-dB SPL calibration standard (Brüel & Kjær Type 4231).

Localisation ability was assessed using a spatial discrimination task. On each trial, the three possible response locations (−60°, 0°, and +60°) were indicated by vertical lines on a 270° visual projection screen. The screen was created by hanging acoustically-transparent fabric directly in front of the loudspeakers. The viewing distance was 1 m and the vertical lines subtended a visual angle of 40°. Participants sat in a chair that was permanently fixed to the floor. A head rest was used to position the head in a consistent position relative to the chair and the screen, and to minimise head movements during stimulus presentation. Participants were instructed to keep their head pointed straight ahead throughout the testing process. A video camera was used to monitor compliance with these instructions. Participants were instructed to listen to each stimulus and to select the location from which the sound appeared to originate. Participants used a trackball to highlight one of the three possible response locations to indicate their response. One location had to be selected and participants were instructed to guess if they were not sure of their answer. An initial warm-up task in which participants listened binaurally confirmed that participants understood the spatial listening task instructions and could respond with 100% accuracy using the trackball.

Stimuli were generated by extracting 150-ms segments at random from the monaural recordings and applying 5-ms onset and offset raised-cosine ramps. The availability of monaural level and spectral cues was manipulated by processing these stimuli prior to presentation. The use of monaural spectral cues was disrupted by filtering the stimuli into ERB_N_-wide channels (Moore, 2012), roving the level of the resulting signals randomly over a 20 dB range in 2-dB increments, and summing the roved signals. This roving range creates considerable uncertainty over the association between spectral content and source location (Wightman and Kistler, 1997) while preserving overall differences in level caused by the head-shadow effect that were informative cues to sound location. Conditions that used these stimuli are referred to as ‘level cue’ conditions. In ‘spectral cue’ conditions, the use of monaural level cues was disrupted by roving the overall presentation level over a 20 dB range in 2-dB increments. The wide roving range disrupted the association between presentation level and location while preserving differences in spectral content that arose between stimuli from the three locations due to interaction with the head and pinna. However, due to the influence of the head-shadow effect on the original monaural recordings, the average level of stimuli originating from the simulated ‘deaf’ side (−60°) when presented to the hearing ear would still have been lower than that of stimuli originating from the hearing side (+60°). While it was unlikely that participants would have been able to detect and exploit this subtle difference due to the wide roving range that was used, the analysis of data from spectral cue conditions accounted for this possibility (see ‘Analysis’).

The factorial combination of the three listening configurations (Unaided, CROS-off, and CROS-on) and two stimulus types (level cue and spectral cue) created six experimental conditions. Participants completed 264 trials in each of these six conditions, resulting in a mean testing time of 14.5 min per condition (range 10.5–22 min). The trials were organised into 8 blocks of 33 trials to allow for an analysis of learning effects within each condition. The blocks always contained 11 trials for each of the three source locations (−60°, 0°, and +60°) and these 11 trials were allocated a unique roving level in conditions where stimulus levels were roved (±10 dB in 2 dB steps).

Performance was assessed across three separate testing sessions that were completed on three different days. A maximum delay of 15 days was permitted between testing sessions. In the first session, performance was assessed separately for the two stimulus types (level cue and spectral cue) while participants listened to the monaural Unaided recordings. The CROS-off and CROS-on listening configurations were completed in the second and third sessions, respectively. The order in which the stimulus manipulations were applied across the 12 participants and across sessions was counterbalanced to account for order effects. The listening configurations were always completed in the same order (Unaided, CROS-off and CROS-on). This approach provided participants with the maximum experience of listening monaurally unaided before their capacity to listen with a CROS device switched on was assessed. This design was adopted to maximise performance in the unaided conditions so as to achieve performance above chance levels and thereby increase the sensitivity of the study to any effects of CROS use on localisation in the horizontal plane.

### 2.6 Training

Participants completed an active training task prior to each of the six experimental conditions. The training was intended to familiarize them with the differing characteristics of sounds across the three possible source locations for each specific combination listening configuration (Unaided, CROS-off, and CROS-on) and stimulus type (level cue and spectral cue). The training also provided repeated exposure to stimuli with feedback that is necessary to learn both level and spectral cues to horizontal localisation (Kumpik et al., 2010). On each trial of the training tasks, a sound was presented from one of the three locations. All three locations were then indicated on the visual projection screen. Participants were instructed to select the location from which they believed the sound had originated. Feedback was then provided by highlighting the actual source location. A total of 30 training trials were completed in each condition prior to the start of testing. The 30 trials provided participants with 10 examples of a stimulus from each of the three sound locations (−60°, 0° and +60° azimuth). The training trials for conditions in which stimulus levels were to be roved provided participants with one example of each unique pairing of location and roving level (−10 dB to +10 dB in 2 dB increments excluding 0 dB).

### 2.7 Analysis

Performance on the spatial discrimination task in each of the six experimental conditions was quantified as the proportion of trials on which the correct location was chosen. An overall performance score was calculated in addition to separate scores for sounds from each of the three source locations. One-sample t-tests were used to compare mean performance levels to chance (33.3% correct).

A repeated-measures ANOVA was used to assess performance in the level cue conditions to determine whether the listening configuration (Unaided, CROS-off, and CROS-on) and source location (−60°, 0° and +60° azimuth) influenced performance. Regression modelling was used to analyse performance in the spectral cue conditions as this approach enabled any effects of presentation level to be partialled out. Two regression models were constructed. A multinomial logistic model assessed whether the responses of participants (i.e. whether they chose −60°, 0°, or +60°) were influenced by the roved presentation level and, critically, whether their responses were related to the source location of the sound once presentation level was controlled for. A binary logistic regression model also assessed whether turning the CROS on had a significant effect on the accuracy of responses once presentation level was controlled for. A Generalised Estimating Equations (GEE) approach using an independence correlation structure was used to account for repeated observations from the same participant.

Binary logistic GEE regression was also used to determine whether switching on the CROS changed the direction and size of the errors that participants made in both the level and spectral cue conditions. Two regression models were constructed. The first model analysed trials on which the actual stimulus came from the centre location (0°) and errors could be directed towards the deaf side or the hearing side. The second model analysed trials on which the actual stimulus came from the deaf or hearing side and on which errors could either be 60° (i.e. participants chose the centre location) or 120° (i.e. participants chose the opposite side). All regression models were computed using the Zelig package (Imai et al., 2008) for the R statistical programming environment (R Core Team, 2016).

## 3 Results

**Fig. 2:**
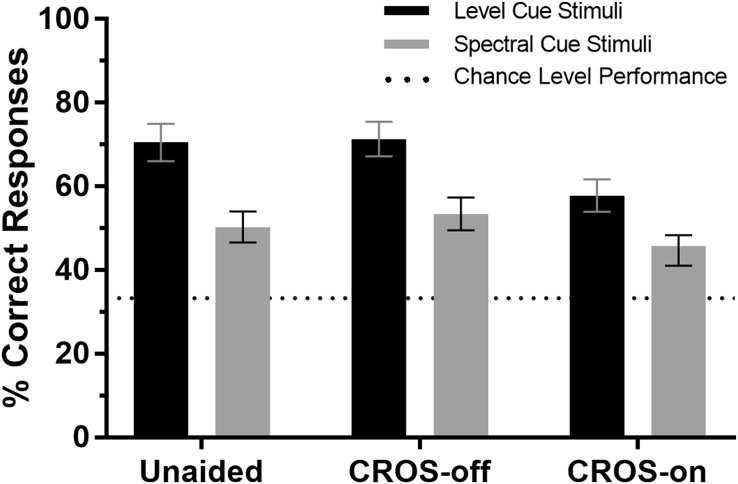
Mean spatial discrimination performance levels in percent correct across the six experimental conditions (defined by three listening configurations and two stimuli types) and their 95% confidence intervals. Chance performance is represented by a horizontal dotted line.

Mean spatial discrimination performance levels and their 95% confidence intervals are shown in Fig. 2. One-sample t-tests confirmed that performance was significantly more accurate than chance (33.3% correct) in all conditions. As predicted, monaural performance levels were similar when the CROS was not worn (unaided) and when the CROS was worn but turned off (CROS-off). Performance levels in the unaided and CROS-off conditions were not statistically-distinguishable (see ‘supplementary material A’ for details of an equivalence analysis). The results from the CROS-off and CROS-on conditions are therefore presented in the following sections (see ‘supplementary material B’ for the results from the unaided conditions).

### 3.1 Analysis of level cue stimuli

**Fig. 3:**
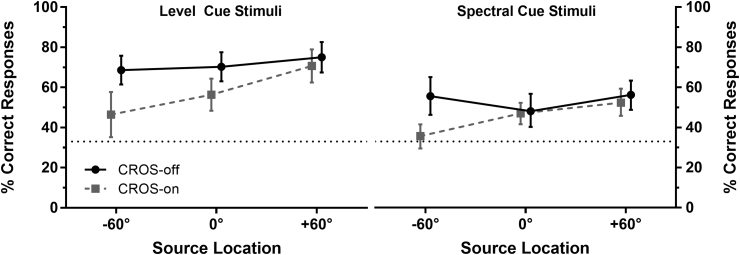
Mean percentage of correct responses for each presentation direction with the CROS turned off (solid lines) and on (dashed lines) for level cue stimuli (left panel) and spectral cue stimuli (right panel). Error bars represent 95% confidence intervals and chance performance is represented by the dotted line.

The left panel of Fig. 3 shows the mean spatial discrimination performance for level cue stimuli at each source location. Performance was found to be more accurate than chance for all source locations in both listening configurations. The data were subjected to a repeated measures ANOVA with within subjects factors of listening configuration (CROS-off vs CROS-on) and presentation direction (−60°, 0° and +60°). Turning the CROS on impaired performance (*F* (1, 11) = 87.50, *p* < 0.001, *η*^*2*^_*p*_ = 0.89) but the degree of impairment was found to vary between source locations (*F* (2, 22) = 4.62, *p* = 0.021, *η*^*2*^_*p*_ = 0.30). Planned contrasts confirmed that relative to the reduction in accuracy for +60° stimuli (5% reduction), turning on the CROS significantly impaired accuracy to a greater extent for −60° stimuli (22% reduction; *F* (1, 11) = 7.52, *p* = 0.019, *η*^*2*^_*p*_ = 0.41) and 0° stimuli (14% reduction; *F* (1, 11) = 7.15, *p* = 0.022, *η*^*2*^_*p*_ = 0.39).

**Fig. 4:**
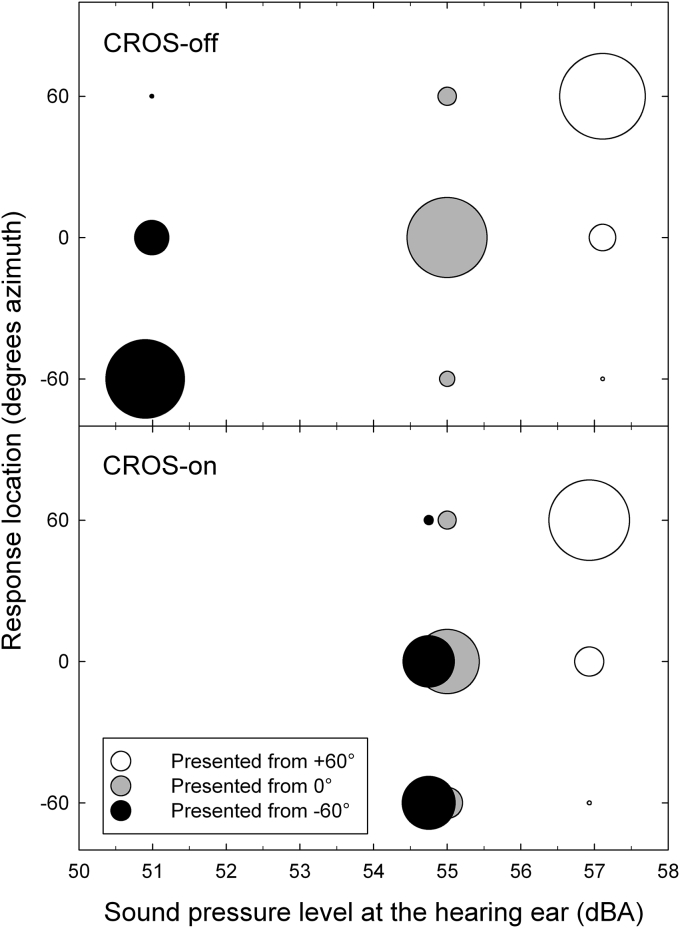
Bubble plot of response choices as a function of source location and presentation level for the level cue stimuli with the CROS turned off (top panel) and turned on (bottom panel). The number of responses is represented by the size of the circle and the source location is indicated by their colour. The sounds pressure level recorded at the hearing ear is represented by the position of the circle on the x-axis in A-weighted dB SPL (dBA).

This variation in spatial discrimination accuracy across source locations can be attributed to the fact that turning the CROS on reduced the difference in level between each pair of source locations in a non-uniform manner. Fig. 4 shows the distribution of responses as a function of both source location and response location when level cue stimuli were presented. With the CROS turned off, spatial discrimination accuracy was similar for stimuli presented from −60°, 0°, and +60° (68%, 70%, and 75%, respectively). With the CROS switched on, the level difference was larger for the +60°/-60° pair (4.4 dB) and the +60°/0° pair (3.9 dB) compared to the −60°/0° pair (0.5 dB). Crucially, this latter difference falls below the 1 dB just-noticeable difference (JND) for changes in amplitude (Middlebrooks and Green, 1991). Therefore, it is to be expected that participants were unable, or at the very least found it very difficult, to discriminate between −60° and 0° stimuli. Hence, turning on the CROS significantly impaired spatial discrimination accuracy for the −60° and 0° source locations by degrading the difference in their levels that was previously a cue to their location.

Logistic regression was used to examine the nature of the detrimental effect that CROS had on spatial discrimination accuracy for the −60° and 0° source locations. For trials on which the stimulus location was 0°, the regression analysis indicated that turning on the CROS significantly increased the likelihood of errors being made towards the deaf side rather than the hearing side (Odds Ratio 2.1, 95% CI 1.0 to 4.1). With the CROS off, 46% of all errors were towards the deaf side and that proportion increased to 63% when the CROS was switched on. An analysis of trials on which the stimulus location was −60° indicated that turning on the CROS significantly increased the odds of erroneously choosing the hearing side rather than the centre location (Odds Ratio 5.3, 95% CI 3.6 to 7.6). However, despite this significant increase only a small proportion of errors were towards the hearing ear (CROS-off 3%, CROS-on 15%).

### 3.2 Analysis of spectral cue stimuli

The right panel of Fig. 3 shows the mean spatial discrimination performance level for spectral cue stimuli at each source location. With the exception of sounds presented from −60° in the CROS-on condition, performance was found to be above chance for all source locations in both listening configurations. Visual inspection of the pattern of responses appeared confirm the expectation that participants' choices would be influenced by the presentation level of the stimuli (see supplementary material C for a figure showing the distribution of response choices as a function of presentation level). Multinomial logistic regression modelling confirmed that participants' response choices in the spectral conditions (independent of whether they were correct or not) were influenced by the roved presentation level of the stimuli (*χ*^*2*^ (1) = 37.8, *p* < 0.001). After the effect of presentation level had been controlled for, responses still varied as a function of the source location of the sound (*χ*^*2*^ (2) = 18.4, *p* < 0.001) suggesting that participants were using information other than the level of the sounds (presumed to be their spectral content) to select a response location.

The binary logistic regression model indicated that accuracy of those responses was not influenced by the presentation level of stimuli (*χ*^*2*^ (1) = 2.4, *p* = 0.125). This result is compatible with the fact that the wide roving range disassociated presentation level and the correct choice of source location. Overall performance was also similar across the three possible source locations (*χ*^*2*^ (2) = 4.4, *p* = 0.113). After effects related to presentation level were controlled for, performance was found to be above chance in both the CROS off (mean accuracy 53.5%, 95% CI 49.5%–57.4%) and CROS on (mean accuracy 44.8%, 95% CI 41.0%–48.4%) conditions. Therefore, not only did spectral cues influence participants' response choices, the cues were also sufficient to support some degree of spatial discrimination with the CROS turned on and off.

Although participants were able to use spectral cues to some extent to determine source location, this ability was significantly impaired when the CROS was turned on (*χ*^2^ (1) = 17.1, *p* < 0.001; mean decrease 8.6%, 95% CI 4.6%–12.6%). The effect of signal re-routing was also found to have varied as a function of source location (*χ*^2^ (4) = 18.7, *p* < 0.001). When sounds came from −60°, performance was less accurate when the CROS was switched on (35.6%) compared to when it was switched off (55.7%) (19.9% decrease, 95% CI 11.1%–28.6%). In contrast, the accuracy with which sounds from 0° to +60° could be identified was similar regardless of whether the CROS was turned off or on (0°: CROS off 48.1%, CROS on 47.0%; +60°: CROS off 56.1%, CROS on 52.3%).

**Fig. 5:**
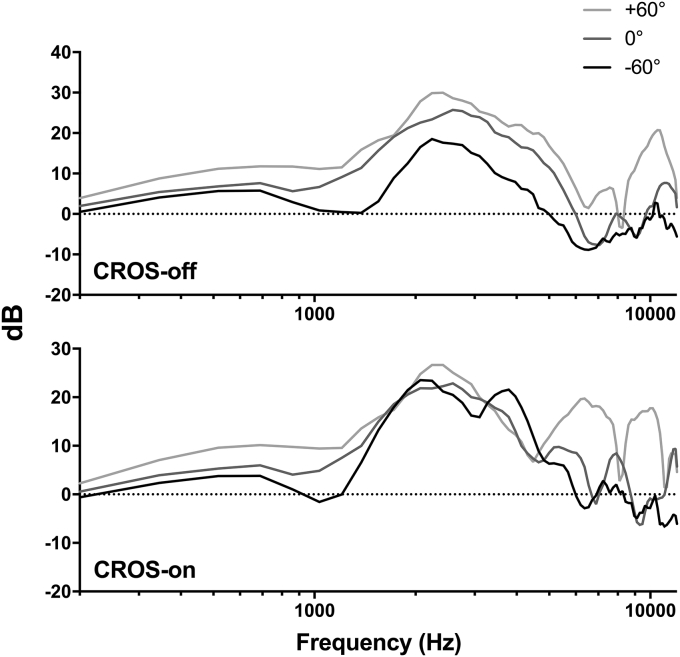
Fast Fourier transforms (FFTs) of the spectral cue stimuli for each source location (−60°, 0° and +60°) with the CROS turned off (top panel) and on (bottom panel).

It is plausible that the degradation in performance observed in the CROS on condition can be attributed to changes in the spectral pattern between the three source locations due to turning the CROS on. Fig. 5 shows the fast Fourier transform (FFT) for stimuli recorded from each source location with the CROS turned off and on. This figure illustrates the CROS induced constriction of spectral differences between the source locations. Note that although turning the CROS on did change the pattern of spectral differences between the source locations (resulting in poorer overall performance driven by fewer correct −60° responses), differences between the locations still existed with the CROS device switched on, particularly at higher frequencies (>5 kHz) where the waveform is small enough to interact with the pinnae to provide spectral cues (Moore, 2012). This partial preservation of spectral differences is likely to be responsible for the above chance performance observed.

## 4 Discussion

The results confirmed the expectations that participants would be able to discriminate above chance levels between sounds from different locations using both monaural level and spectral cues (hypothesis 1), that under carefully controlled conditions with an adequately-powered sample it would be possible to demonstrate detrimental effects of CROS use on localisation in the horizontal plane (hypothesis 2), and that these effects were due to the re-routing of signals rather than any occlusion of the hearing ear (hypothesis 3). The results also demonstrate how CROS use affects the availability and use of monaural level and spectral cues to horizontal sound location, which had not been examined previously (Kitterick et al., 2016).

CROS disrupted access to monaural level cues by overcoming the head shadow effect; that is, by minimizing the difference in level between sounds originating on the deaf and hearing sides of the head (Pumford, 2005). The confirmation of this detrimental effect is particularly unfortunate as both the current study and previous research has demonstrated that monaural listeners show a preference to use level cues over spectral cues and are more accurate at discriminating source locations in the horizontal plane when using level cues (van Wanrooij and van Opstal, 2004). The CROS fitting process therefore represents a trade-off between maximising access to sound on both sides of the head (its intended effect) and preserving differences in level that can be a useful cue to sound location.

The current study also found evidence that CROS disrupts access to what are referred to here as ‘spectral cues’. The use of spectral cues typically refers to the exploitation of high-frequency pinnae cues to determine sound source elevation and to resolve front-back confusions (Wightman and Kistler, 1997). In the present context, they refer to spectral information that monaural listeners can exploit to achieve above-chance levels of localisation in the horizontal plane (Shub et al., 2008, Kumpik et al., 2010, Rothpletz et al., 2012). The capacity of monaural listeners to use spectral cues for vertical and horizontal localisation has been observed to be highly correlated, possibly indicating that similar stimulus features may underpin both abilities, and that the use of spectral information for horizontal localisation can be of limited use outside of the hearing hemifield (van Wanrooij and van Opstal, 2004). The precise mechanism that drives the ability to utilise spectral cues to localise in the horizontal plane remains unclear, but in the absence of binaural interaural cues the auditory system appears to be able to learn to distinguish between spectral cues that code elevation from those that code position in the horizontal plane (Agterberg et al., 2014). The process of learning to use spectral information for horizontal localisation has been shown to require repeated exposure to stimuli with stable and predictable spectral content (Kumpik et al., 2010).

Although CROS impeded the ability to localise both level and spectral cue stimuli, performance was significantly poorer in the spectral cue conditions relative to the level cue conditions across every listening condition (Fig. 2). In fact, the best performance observed for spectral cue stimuli (CROS off) was still poorer than the worst performance observed for level cue stimuli (CROS on). The limited accuracy with which participants could use spectral cues arose despite the use of a testing methodology that aimed to maximise their use; i.e. by constraining the spectral content of the sound source to be constant across trials (Kumpik et al., 2010) and using only a limited number of source locations (−60°, 0°, and +60°). Numerous studies have documented the difficulties that listeners have with using monaural spectral cues to determine source location (Jin et al., 2004, Rothpletz et al., 2012). However, it is perhaps encouraging that CROS use does not appear to completely remove the availability to use level and spectral cues given that they can be exploited by at least some experienced monaural listeners (Newton, 1983, Shub et al., 2008).

The present findings point to a detrimental effect of CROS on horizontal sound localisation that was consistent in magnitude and direction across individuals (all participant scores decreased with CROS use). This conclusion contrasts with the inconsistent nature of the evidence from clinical studies of the effect of CROS on sound localisation in monaural listeners (for systematic reviews see Peters et al., 2015, Kitterick et al., 2016). The extent to which CROS disrupted monaural cues to sound location may have varied across these clinical studies due to a wide range of factors including differences in the choice of stimuli (with particular reference to the decoupling of level and spectral cues), the choice and number of source locations, the use of discrete or continuous response methodologies including the use of pointing methods, whether the source locations were visible or not, and the size of the roving range applied. For example, the use of a small number of loudspeakers as in the current study would be expected to minimise the difficulty of the task when listening monaurally and therefore maximise the size of any detrimental effects of CROS use. There may also have been differences in the methodology used to fit the CROS device; the current study used Real Ear Measurements (REMs) to verify the CROS fitting according to an established protocol (Pumford, 2005). It is possible that the prescriptions in clinical studies will have been based solely on a patient's audiogram and not verified using REMs, the so-called ‘click-and-fit’ approach. If so, that approach would have been unlikely to minimise the availability of monaural cues consistently and to the extent achieved in the current study due to variability in the size of the head shadow effect across individuals. Finally, by simulating monaural hearing in binaural subjects with normal hearing thresholds, the current study minimised variability in high frequency hearing loss, which can affect monaural localisation abilities (Agterberg et al., 2014).

Monaural listeners place a high value on restoring sound localisation, rating it second in importance only to speech perception in noise (McLeod et al., 2008). The present findings confirm that CROS does not improve localisation in the horizontal plane under ideal fitting and testing conditions and in fact impedes localisation. An immediate implication of this research is to ensure that patients' expectations are managed about the intended purpose of CROS devices; that is, to improve access to sound on the side of the deaf ear. Setting appropriate expectations could avoid non-use due to a lack of benefit to sound localisation. It could also be useful to characterise the spatial listening abilities of a monaural listener prior to CROS fitting as previous work has shown a high degree of inter-individual variability (Agterberg et al., 2014, Van Wanrooij and van Opstal, 2004). Those patients who demonstrate an ability to use monaural cues may require a CROS prescription that trades some access to sound on the deaf side for access to level differences between sounds on either side of the head. For all patients, the information provided about the CROS device should serve to make them aware of the potential deleterious effects on spatial listening and empower them to determine when it may be appropriate to use it and when its use may be counterproductive.

### 4.1 Summary

The present results demonstrate that CROS use disrupts the availability of monaural level and spectral cues to localisation in the horizontal plane. Ultimately, accurate sound localisation requires two functioning ears and existing solutions to restore binaural hearing, such as cochlear implantation, are invasive and expensive and are not available or indicated for all patients. The difficulties that impaired spatial hearing creates and the importance that unilaterally-deaf patients place on restoring their ability to localise are strong motivators for developing novel CROS fitting methodologies that minimise these deleterious effects or alternative interventions that could support some level of spatial awareness and localisation ability in individuals with only one functioning ear.

## Funding

This work was supported by the intermural funding of the Medical Research Council, Institute of Hearing Research [research code RA4877]; and infrastructure funding from the National Institute for Health Research [NOTHEBRU-2012-1]. The views expressed are those of the authors and not necessarily those of the NHS, NIHR, or the Department of Health.

## Contributions

The study was conceived by PK and designed by AP and PK. AP collected the data. AP and PK analysed the data, interpreted the findings, and wrote the manuscript.

## Figures and Tables

**Figure:**
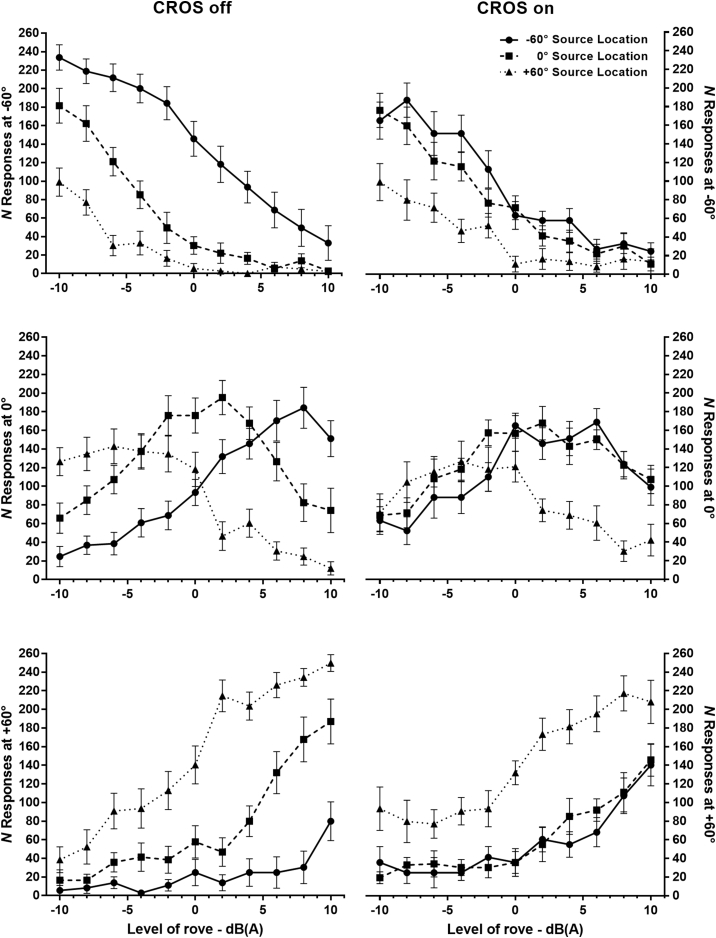


## Appendix A Supplementary data

Supplementary material A: Analysis of statistical equivalence between spatial discrimination performance in the Unaided and CROS-off conditions conducted to confirm the hypothesis that monaural localisation performance would be similar in both listening configurations.

Supplementary material B: Numerical data behind manuscript Fig. 2, Fig. 3, Fig. 4.

Supplementary material C: Figure showing the mean number of times participants chose each response location (top panels: −60°, middle panels: 0° bottom panels +60°) when the was CROS turned off (left panels) and on (right panels) as a function of roving level. Within each panel, response curves are plotted separately for each of the three source locations (solid lines: −60°, dashed lines: 0°, dotted lines: −60°). Error bars plot 95% confidence intervals.
