# Supplementary material for: Contralateral routing of signals disrupts monaural level and spectral cues to sound localisation on the horizontal plane
Source: Hear Res. 2017 Sep;353:104–11. doi: 10.1016/j.heares.2017.06.007 (PMC5603973; doi:10.1016/j.heares.2017.06.007)
Supplement: Supplementary file 1 [file mmc1.docx]

*Supplementary Material A: Analysis of equivalence between the Unaided and CROS-off conditions.*

An equivalence analysis was conducted to confirm the hypothesis that monaural localization performance would be similar when listening to the Unaided and CROS-off recordings. The ‘two one-sided tests’ (TOST) test was used to assess equivalence. To inform this analysis, it was necessary to define the smallest change in performance that would be considered meaningful. In the absence of a definition of what constituted a meaningful change on the spatial discrimination task that could be specified *a priori*, the smallest detectable change that could be reliably detected by the task (SDC) was used as the threshold for equivalence.

The SDC was calculated using a three-step process. First, intra-class correlation coefficients (ICC) were calculated between performance in the first and second halves of other available monaural conditions (CROS-on spectral cue ICC: 0.79; CROS-on level cue ICC: 0.90). Second, these ICC values and the standard deviation within the first half of each condition (spectral cue 6.3%, level cue 6.6%) were used to derive an estimate of the standard error of measurement (SEM: spectral 2.9%, level 2.1%). Third, these SEM values were used to determine the SDC; i.e. the smallest change in accuracy that was reliably detectable. The SDC was estimated to lie between 5.7% (CROS-on, spectral cue conditions) and 8.0% (CROS-on, level cue conditions). The more conservative of these two estimates (5.7%) was used as the threshold for the equivalence analysis. Using this threshold, TOST tests confirmed that performance was equivalent in the unaided and CROS-off conditions for both the spectral cue conditions (mean difference -1.8%, 95% CI -1.4% to 5.0%) and level cue conditions (mean difference 1.5%, 95% CI -1.3% to 4.4%). Thus, wearing the CROS device while switched off did not influence localisation accuracy in a manner that could be distinguished from measurement error.
