# Supplementary material for: Contralateral routing of signals disrupts monaural level and spectral cues to sound localisation on the horizontal plane
Source: Hear Res. 2017 Sep;353:104–11. doi: 10.1016/j.heares.2017.06.007 (PMC5603973; doi:10.1016/j.heares.2017.06.007)
Supplement: Supplementary file 2 [file mmc2.docx]

*Supplementary Material B: Performance levels in the unaided conditions not shown graphically in Figures 3 and 4.*

Table 1: Mean percentage of responses, SD and 95% CI’s for each stimuli manipulation (level cue and spectral cue) and source location (-60°, 0° and +60°) combination in the unaided conditions.

| Stimuli Type | Source Location | Mean (%) | SD |
| --- | --- | --- | --- |
| Level | -60° | 68.6 | 9.8 |
| Level | 0° | 68.9 | 17.0 |
| Level | +60° | 74.0 | 12.7 |
| Spectral | -60° | 50.5 | 6.1 |
| Spectral | 0° | 45.2 | 4.5 |
| Spectral | +60° | 54.7 | 4.8 |

Table 2: Mean percentage of responses for each source location (-60°, 0° and +60°) and response direction (-60°, 0° and +60°) combination for level cue stimuli in the unaided conditions.

| Source location | -60°  Responses | 0°  Responses | +60°  Responses |
| --- | --- | --- | --- |
| -60° | 68.6% | 28.6% | 2.75% |
| 0° | 13.5% | 68.9% | 17.6% |
| +60° | 2.1% | 24.0% | 74.0% |
